# Resolving the natural myocardial remodelling brought upon by cardiac contraction; a porcine ex-vivo cardiovascular magnetic resonance study of the left and right ventricle

**DOI:** 10.1186/s12968-019-0547-2

**Published:** 2019-07-01

**Authors:** Camilla Omann, Peter Agger, Nikolaj Bøgh, Christoffer Laustsen, Steffen Ringgaard, Robert S. Stephenson, Robert H. Anderson, Vibeke E. Hjortdal, Morten Smerup

**Affiliations:** 1 Department of Cardiothoracic & Vascular Surgery, Aarhus University Hospital, Skejby, Denmark; 2 Department of Clinical Medicine, Aarhus University, Palle Juul-Jensens Boulevard 99, 8200 Aarhus N, Denmark; 3 Comparative Medicine Lab, Aarhus University Hospital, Skejby, Denmark; 4 MR Research Centre, Aarhus University, Aarhus, Denmark; 5 Institute of Clinical Sciences, College of Medical and Dental Sciences, The University of Birmingham, Birmingham, UK; 6 Institute of Genetic Medicine, Newcastle University, Newcastle-upon-Tyne, UK

**Keywords:** Myocardium, Diffusion tensor imaging, Micro-structure, Dynamic rearrangement, Myocyte orientation, Myocardial architecture

## Abstract

**Background:**

The three-dimensional rearrangement of the right ventricular (RV) myocardium during cardiac deformation is unknown. Previous in-vivo studies have shown that myocardial left ventricular (LV) deformation is driven by rearrangement of aggregations of cardiomyocytes that can be characterised by changes in the so-called E3-angle. Ex-vivo imaging offers superior spatial resolution compared with in-vivo measurements, and can thus provide novel insight into the deformation of the myocardial microstructure in both ventricles. This study sought to describe the dynamic changes of the orientations of the cardiomyocytes in both ventricles brought upon by cardiac contraction, with particular interest in the thin-walled RV, which has not previously been described in terms of its micro-architecture.

**Methods:**

The hearts of 14 healthy 20 kg swine were excised and preserved in either a relaxed state or a contracted state. Myocardial architecture was assessed and compared between the two contractional states by quantification of the helical, transmural and E3-angles of the cardiomyocytes using high-resolution diffusion tensor imaging.

**Results:**

The differences between the two states of contraction were most pronounced in the endocardium where the E3-angle decreased from 78.6° to 24.8° in the LV and from 82.6° to 68.6° in the RV. No significant change in neither the helical nor the transmural angle was found in the cardiomyocytes of the RV. In the endocardium of the LV, however, the helical angle increased from 35.4° to 47.8° and the transmural angle increased from 3.1° to 10.4°.

**Conclusion:**

The entire myocardium rearranges through the cardiac cycle with the change in the orientation of the aggregations of cardiomyocytes being the predominant mediator of myocardial wall thickening. Interestingly, differences also exist between the RV and LV, which helps in the explanation of the different physiological capabilities of the ventricles.

**Electronic supplementary material:**

The online version of this article (10.1186/s12968-019-0547-2) contains supplementary material, which is available to authorized users.

## Background

Structural remodelling of the myocardium has been attributed to the functional impairment observed in heart disease [1] and cardiac anatomy is, therefore, the foundation of both normal and abnormal cardiac function. But despite centuries of research, the micro-anatomy of the heart and its intricate relationship with cardiac function is still debated. Further detailed knowledge of how micro-structural changes facilitate wall deformation during the cardiac cycle is, therefore, essential for improving the understanding of cardiodynamics in health and disease [2].

Overall, the right ventricle (RV) and left ventricle (LV) serve the same purpose of pumping blood against an afterload, but apart from this they differ in most other aspects. Their anatomy and mural thickness is far from identical, and the same is true of their physiology [3, 4], biochemistry [5] and embryological origin [6]. They also respond differently to changes in haemodynamic load [7]. It could be argued that the functional difference between the two ventricles is caused by the difference in myocardial wall-thickness, but neither the naturally ‘hypertrophied’ RV after birth nor the pathologically hypertrophied RV can cope with changes in afterload conditions in the same way as can the LV. When the two ventricles are subjected to similar changes in afterload the RV is more likely to fail [4].

Although there are considerable data on the mechanisms of LV dysfunction and failure, the pathophysiological mechanisms underlying RV dysfunction and failure are only beginning to be understood [7]. Heart failure differs greatly in terms of etiology and prognosis depending on which ventricle is affected. LV heart failure often occurs in adulthood and is most commonly caused by myocardial ischemia, systemic hypertension and aortic valve stenosis. RV heart failure, on the other hand, is often related to congenital heart disease [7]. Today heart failure remains a clinical diagnosis. One reason for this is that the pathophysiological mechanisms underlying heart failure are still not fully understood. Changes in the structure of the myocardium and the associated loss of function are major contributing factors to heart failure [1, 8]. These factors, therefore, have a prognostic potential in relation to cardiac diseases [9], but crucially we are yet to understand the structural mechanisms underlying RV contraction.

We know ventricular wall deformation coincides with contraction of the cardiomyocytes, however, cell shortening alone is not sufficient to produce normal ejection fractions [10]. Cardiac deformation is achieved, therefore, by dynamic rearrangement of the components of the ventricular walls. The myocardium composes a complex heterogeneous three-dimensional meshwork of cardiomyocytes exhibiting both helical and transmural orientations. The myocytes are, moreover, aggregated into groups often referred to as units, sheets or sheetlets, which are embedded in a collagen framework [11–13]. Because the anatomical extent of such aggregates is still unknown we chose to abstain from referring to them by any other name but aggregates. Using diffusion tensor cardiovascular magnetic resonance (CMR), the cardiac mesh has been characterised according to the helical and transmural orientation of the cardiomyocytes [14–16]. It has been demonstrated mathematically that the distribution of helical and transmural angles is necessary to equalise and normalise systolic myocyte strains to a physiological range [10, 11, 17]. This suggests an intimate link between orientation of the cardiomyocyte chains and physiological deformation of the myocardium and highlights the necessity for significant spatial rearrangement of the cardiac mesh from diastole to systole.

Rearrangement of the myocardial architecture through the cardiac cycle has been demonstrated previously using in-vivo diffusion tensor magnetic resonance imaging in the LV of both healthy [2, 18] and diseased hearts [19, 20]. Recent studies of the LV myocardium identify, not only changes in myocyte orientation, but also the orientation of aggregated cardiomyocytes, as an important contributor to wall deformation [20, 21]. Despite this, the low spatial resolution inherent of in-vivo diffusion tensor CMR, namely 1x1x8 mm^3^, means the intricacies of wall deformation cannot be resolved and neither can the morphological details of the relatively thin walled RV. Questions remain regarding regional and transmural differences in deformation and the functional significance of the aggregations of cardiomyocytes. Ex-vivo imaging offers superior spatial resolution, and as a result, can provide novel insight into the rearrangement of all components of the cardiac mesh relative to cardiac phase, region, and wall depth in both the LV and RV.

## Methods

In the present study, using high resolution ex-vivo diffusion tensor CMR, we describe the changes in orientation of the cardiac mesh components in the LV and RV, and muscular ventricular septum, brought upon by cardiac contraction.

### Animal experiments

We included 14 Danish landrace female swine in the study each weighing 20 kg. All experiments were approved by the Danish Animal Inspectorate license no. 2013-15-2934- 00869.

Prior to experiments, the swine were kept in their usual farm environment, with unrestricted access to food and water. Artificial daylight was maintained for 12 h, from 8 am to 8 pm. Before transportation to the experimental laboratory, the swine were pre-anesthetised with intramuscular administration of 0.5 mg/kg of midazolam and 2.5 mg/kg of ketaminol. If needed this was repeated upon arrival to the laboratory to allow establishment of an intravenous access. Anaesthesia was supplemented by intravenous administration of 3 mg/kg of propofol to allow endotracheal intubation and coupling to a ventilator. Continuous anaesthesia was maintained using 2–3% inhalational sevoflurane. While anaesthetised a venous 6 French sheath was placed in the femoral vein using Seldinger technique guided by ultrasound.

The animals were then transferred to our CMR facilities to perform CMR for determination of overall cardiac anatomy. To achieve maximal myocardial contraction within the normal physiological range, intravenous administration of dobutamine 40 μg/kg/min was introduced in order to simulate an exercise situation. The animals were then returned to our experimental operating theatre where their hearts were exposed through a median sternotomy while still fully anaesthetised. After intravenous administration of 10.000 units of heparin, the animals were euthanised by means of excision of the heart and great vessels. The hearts were subsequently randomised into fixation in either a relaxed state or a contracted state. In the group randomized to the relaxed state, 7 hearts were each perfused with one litre of potassium rich cold cardioplegic solution (Kardioplex; H/S Apoteket, Copenhagen, Denmark) immediately after excision directly through the coronary arterial orifices at a pressure of approximately 100 mmHg at the point of the catheter tip. Subsequently, a water-based CMR compatible polymer (Histomer) was injected into the ventricles via the atrioventricular valvar orifices to maintain the relaxed state. Residual polymer could escape via the pulmonary and aortic valvar orifices to avoid excess dilation. The polymer was allowed to harden for approximately 15 min, after which the hearts were perfused with formalin 10% (Lillies fluid, pH 7.4) using the same method as with the cardioplegic solution. In the group randomized to contraction, 7 hearts were perfused with formalin immediately after excision of the hearts. No polymer was injected in this group. All hearts were kept in formalin for at least 48 h to ensure complete transmural fixation.

### Imaging sequences

#### Cardiovascular magnetic resonance imaging

CMR was performed in a 1.5 T CMR scanner (Achieva dStream, Philips Healthcare, Best, Netherlands). The animals were placed on the scanner bed in a supine position and anaesthesia was maintained using 2–3% inhalational sevoflurane. Scout images determined the orientation of the LV long-axis. LV function was assessed using a retrospective, electrocardiogram-triggered balanced steady state free precession (bSSFP) breath-hold cine sequence in the cardiac short-axis, vertical long-axis and horizontal long-axis plans. In the cardiac short-axis, LV volume was completely encompassed by contiguous 8 mm slices with a spatial resolution of 1.22 mm × 1.22 mm and a field of view of 288 mm × 288 mm. The following imaging parameters were used: repetition time 3.0 ms; echo time 1.5 ms; flip angle 60°; 30 heart phases. Heart rate and blood pressure was continuously recorded.

#### Diffusion tensor cardiovascular magnetic resonance imaging

Twenty-four hours prior to diffusion tensor CMR scanning, the hearts were perfused with phosphate buffered solution and stored at 4 °C. On the day of diffusion tensor imaging the hearts were allowed to adapt to room temperature before initiating the scan. Imaging was performed using an Agilent 9.4 T MR-system (Agilent, Santa Clara, California, USA), equipped with 400 mT gradients and vnmrJ 4.0 software. Each heart was placed with LV long-axis aligned parallel to the axis of the main magnetic field. Scout imaging enabled precise adjustment of axis and rotation of the images. Measurements were performed using a standard multi-slice 2D spin echo sequence with diffusion gradients. The repetition time was 7000 ms and echo time was 30 ms. The scan time was approximately 16 h for each heart. We used 30 isotopically distributed diffusion directions with a b-value of 1000 s/mm^2^ and one additional with b = 0 s/mm^2^, 125 slices with 800 μm slice thickness and no gap and an in-plane-resolution of 400 × 400 μm^2^.

### Assessment of myocardial contraction

**Fig. 1:**
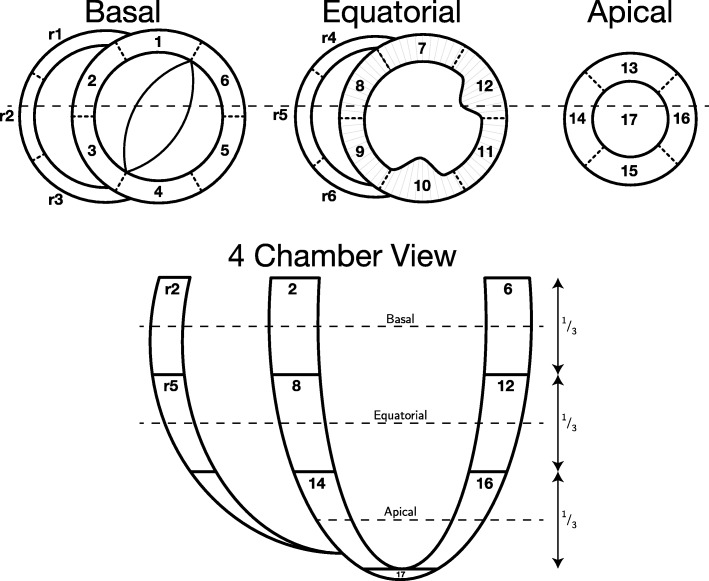
Myocardial zones. Schematic representation of the cardiac zones adapted from Agger et al. [22]. *Left ventricle*: 1, basal superior; 4, basal inferior; 5, basal inferolateral; 6, basal superolateral; 7, equatorial superior; 10, equatorial inferior; 11, equatorial inferolateral; 12, equatorial superolateral; 13, apical superior; 14, apical septal; 15, apical inferior; 16, apical lateral; 17, apex. 64 sections of equal size are illustrated in the left ventricle in the equatorial plane. *Septum:* 2, basal superoseptal; 3, basal inferoseptal; 8, equatorial superoseptal; 9, equatorial inferoseptal. *Right ventricle*: r1, basal anterosuperior; r2, basal anterior; r3, basal anteroinferior; r4, equatorial anterosuperior; r5, equatorial anterior; r6, equatorial anteroinferior

To evaluate the state of contraction of the excised hearts, ventricular wall thickness from the ex-vivo diffusion tensor imaging data, was compared with that of the in-vivo CMR cine data. In order to do so the ventricular myocardium was virtually subdivided into 23 zones as previously described in our group and illustrated in Fig. 1 [22]. All wall thickness measurements were done in the equatorial level. To avoid potential disturbances from papillary muscles, LV thicknesses were assessed only in zones 7 and 11.

Septal measures were taken in zones 8 and 9 whereas zone r4, r5 and r6 quantified the RV wall thickness. Mean wall thickness was measured in-vivo in end-diastole and end-systole both before and after dobutamine administration. All image analyses of in-vivo data were done manually using Segment version 2.0 R4942 (http://medviso.com/segment/) while determination of the ex-vivo wall thickness was done on the diffusion-weighted images using an investigator independent mathematica algorithm measuring the distance between the most epicardial voxel and the most endocardial voxel in the centre of the zones of interest. Ventricular mural thickness was used rather than ventricular volumes in order to reduce the risk of type two error when comparing the low-resolution in-vivo CMR data with the high-resolution ex-vivo data.

### Assessment of myocardial architecture

#### Initial data analyses

Using custom-made software [14, 22], we calculated the diffusion tensors and the corresponding primary, secondary and tertiary eigenvectors of each voxel within the myocardium. These vector data were subsequently imported into Mathematica 10 (Wolfram Research, Inc., Champaign, Illinois, USA (2016)). Data were rotated relative to the long-axis of the LV as defined by a line between the apex of the LV and the aorto-mitral fibrous continuity. Each data set was subdivided into the aforementioned 23 zones [22]. Zones 1, 4, 10, 12 and 17, being the zones including the two interventricular hinge points, the papillary muscles, and the apical vortex, were excluded to avoid potentially contentious areas. The heart was then virtually subdivided into three short axis regions; the basal region, the equatorial region and the apical region (Fig. 1). The circumference of the LV was subdivided into 64 sections of equal size (Fig. 1). The RV was likewise subdivided into sections of similar size as the LV, however, the number of sections varied depending on the size of the ventricle. Within each section all angles were calculated relative to the local epicardial tangential plane as described previously [8]. On average, we analysed approximately 20,200 voxels in each heart.

#### Helical, transmural and E3-angles

**Fig. 2:**
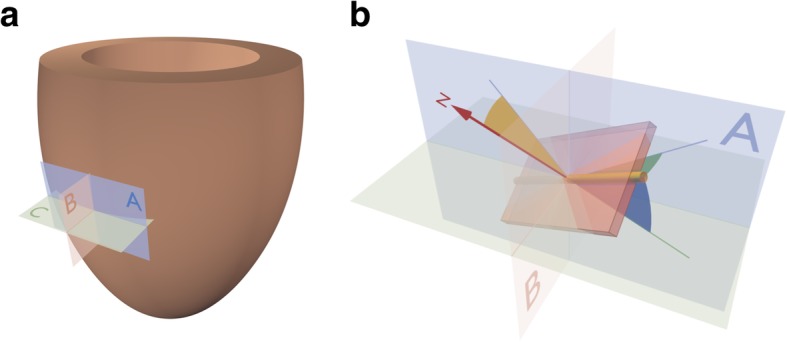
Angle definitions. **a**: The local set of orthogonal planes for the assessment of cardiomyocyte orientation. The plane A is the local epicardial tangential plane. Plane B is the radial-longitudinal plane parallel to the left ventricular long axis and orthogonal to plane A. Plane C is defined as the plane spanned by normal of plane A and the local epicardial horizontal (short axis) tangent. **b**: The helical angle (blue) is defined as the angle between the cardiomyocytes (yellow rod) and plane C. The transmural angle (green) is defined as the angle between the cardiomyocytes and the epicardial tangential plane A. The E3-angle (yellow) is defined as the angulation between the normal (N) of the aggregate plane (box) and the epicardial tangential plane A. A three-dimensional rotatable 3D PDF of panel **b** can be found in the supplementary material as Additional file 1

As in our previous publications, the helical angle was defined as the angle between the primary eigenvector and the local short axis plane C in Fig. 2. As generally accepted in literature, we defined right-handed helical orientations to be positive and left-handed helical orientations to be negative [23]. The transmural angle [16, 24, 25] was defined as the angle between the primary eigenvector and the epicardial tangential plane; this angle thus representing the deviation of the primary eigenvector away from a tangential or surface parallel orientation (Fig. 2). A rotatable 3D PDF version of Fig. 2 is available online as Additional file 1. The tertiary eigenvector is generally accepted to represent the normal vector of the plane of aggregated cardiomyocytes [13, 19, 25]. To assess the orientation of the aggregations we, therefore, measured the angulation between tertiary eigenvector and the epicardial tangential plane as the E3-angle [8, 22]. In this way positive and negative angles represent the previously described two populations of aggregates where the extremes ±90° are parallel to the epicardial surface and 0° is strictly transmural.

### Statistical analysis

Normality was tested using Q-Q plots and histograms. All statistical tests presumed a significance level of 5%. Myocardial wall thickness data were compared between groups using Students t-test. Within each zone all angle values were binned relative to 10 myocardial levels. In each bin and for each angle type the median value was calculated. Zones 5–7, 11, 13–16 were grouped representing the entire LV where as r1 through r6 were grouped representing the RV. Lastly, zone 2, 3, 8 and 9 were grouped as the septum.

Owing to the lack of an appropriate non-parametric alternative, two-way ANOVA was used for comparing overall differences between groups, as this regime has been proven robust also with non-parametric data [26]. In case of significant differences between groups post-hoc testing was performed using Mann-Whitney U-test with Bonferroni correction for multiple comparisons. E3-angle data are most appropriately considered as ‘circular’, because an aggregate with an E3-angle of − 90° has the same radial orientation as one with an angle of + 90°. For the purpose of describing and comparing these data, circular statistics has been applied [27]. Variance analysis was not performed on circular data; instead these data were compared on a point-by-point fashion using Mann-Whitney U-test with Bonferroni correction for multiple comparisons. Stata Statistical Software, release 11 (StataCorp LP, College Station, Texas, USA) and Mathematica 10 (Wolfram Research, Inc.) were used for statistical analyses.

## Results

### Assessment of cardiac phase

**Fig. 3:**
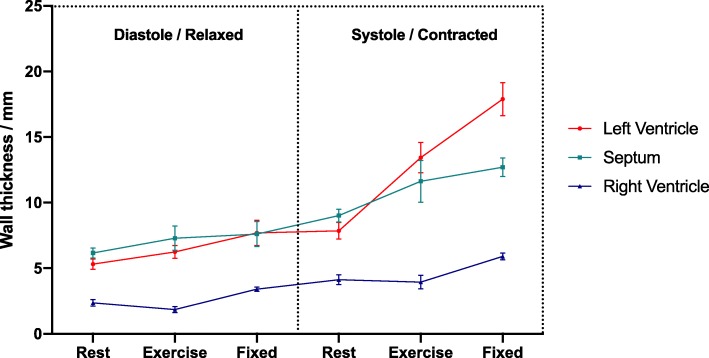
Myocardial wall thickness. Myocardial septal wall thickness. In-vivo data was assessed at rest and during dobutamine stress mimicking physiological exercise. After excision and fixation, the hearts were scanned ex-vivo. Data represented as means with confidence interval as error bars

In-vivo and ex-vivo septal myocardial wall thickness measurements are illustrated in Fig. 3.

Figure 3 shows a significant difference in wall thickness between the hearts fixed in the relaxed state and the hearts fixed in the contracted state in the LV (*p* <  0.001), the septum (*p* <  0.001) and the RV (*p* <  0.001), confirming fixation in two significantly different contractional states (Table 1).

**Table 1:** Ex-vivo wall-thickness for the left ventricle, interventricular septum and the right ventricle compared between relaxation and contraction

	Wall Thickness Relaxation (mm)	Wall Thickness Contraction (mm)	*P*-value
Left Ventricle	7.2 (1.5)	14.4 (2.7)	< 0.001
Septum	7.8 (0.9)	13.2 (1.5)	< 0.001
Right Ventricle	3.8 (0.3)	6.2 (0.9)	< 0.001

Data are presented as mean (SD)

In the LV, no significant change was found between the mean diastolic exercise wall thickness of 6.24 mm (95% CI: 5.8;6.7) and the mean wall thickness in the ex-vivo relaxed state of 7.7 mm (95% CI: 6.7;8.7), *p* = 0.074. Likewise, no significant difference was found between the corresponding values in the septum of 7.3 mm (95% CI: 6.5;8.1) during exercise and 7.6 mm (95% CI: 7.2;8) in fixation, *p* = 0.4. In the RV, a significant difference was found between the diastolic exercise wall thickness of 1.9 (95% CI: 1.6;2.1) and the mean wall thickness in the ex-vivo relaxed state of 3.4 (95% CI: 3.2;3.6), *p* <  0.0001.

No significant difference was found between the mean systolic septal wall thickness during exercise of 11.6 (95% CI: 10;13.2) and that in the ex-vivo contracted state of 12.7 (95% CI: 12;13.4), *p* = 0.14. In the LV, a significant difference was found between the mean systolic wall thickness during exercise of 13.4 (95% CI: 12.3;14.6) and the fixed contracted wall thickness of 17.9 (95% CI: 16.6;19.1), *p* <  0.001. In the RV, a significant difference was found between the systolic exercise wall thickness of 3.9 (95% CI: 3.4;5.6) and the fixed contracted wall thickness of 5.9 (95% CI: 5.6;6.2), *p* <  0.001.

### Assessment of wall deformation by diffusion tensor imaging

**Fig. 4:**
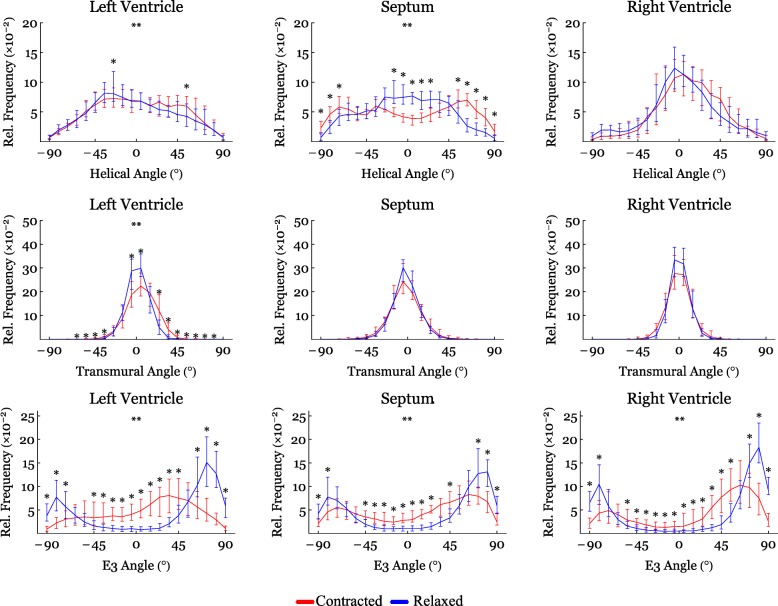
Angle distributions. Histograms of the distributions of myocyte angulations from relaxation to contraction. Data are shown as medians (Interquartile Range). **: Statistically significant overall two-way ANOVA. *: Statistical significance in the individual area derived from post-hoc testing with Mann-Whitney U-Test with Bonferroni correction

**Fig. 5:**
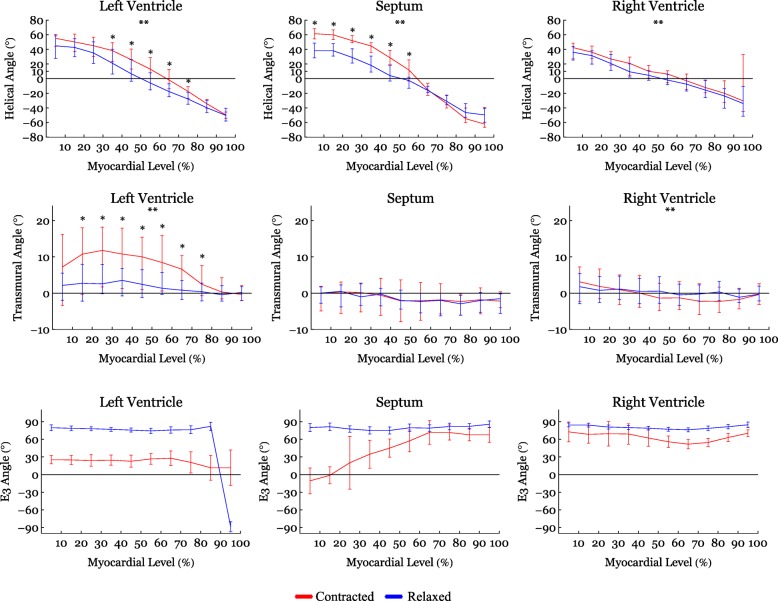
Angles versus myocardial depth. Helical, transmural and E3-angles as a function of myocardial depth in percent. 0% is the sub-endocardium, 100% is the sub-epicardium. In the interventricular septum 100% is the right ventricular sub-endocardium. Helical and transmural angles are shown as medians (Interquartile Range) while E3-angles are shown as circular means (CI). **: Statistically significant overall two-way ANOVA. *: Statistical significance in the individual area derived from post-hoc testing with Mann-Whitney U-Test with Bonferroni correction

**Fig. 6:**
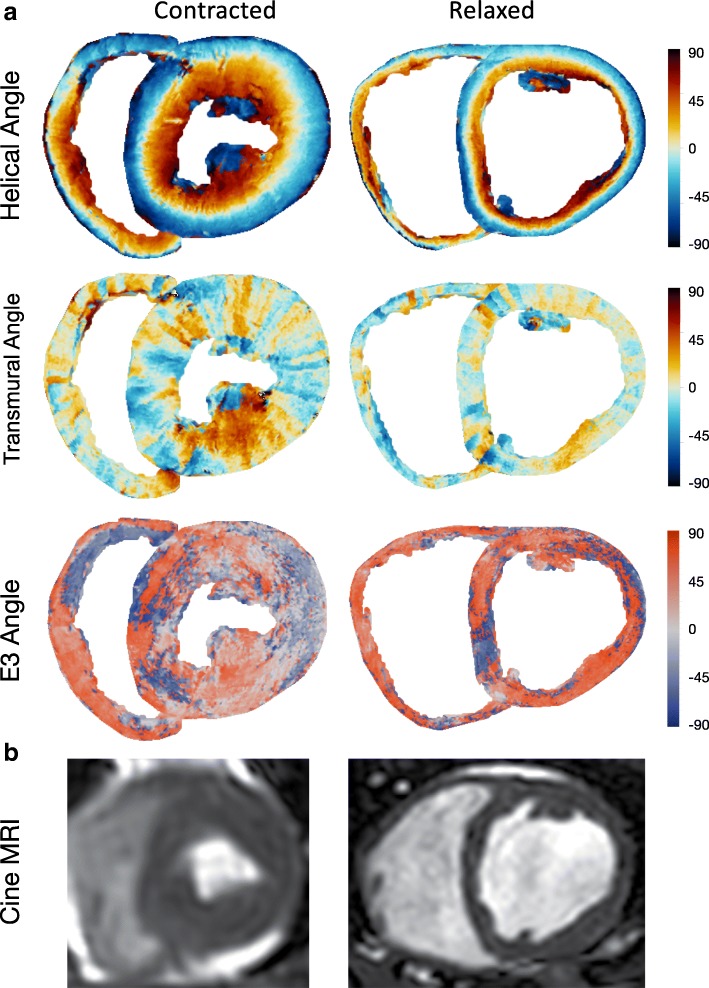
Angle plots. **a**: Representations of the basal plane of a systolic and a diastolic heart. Top three rows are surface plots of helical, transmural and E3-angles. The colors represent the angles illustrated by the panel to the right. **b**: Bottom row are images of in-vivo cardiac cineMRI short axis images of the same hearts. The systolic image is slightly blurred as a result of motion artifacts due to high heart rate during exercise

**Fig. 7:**
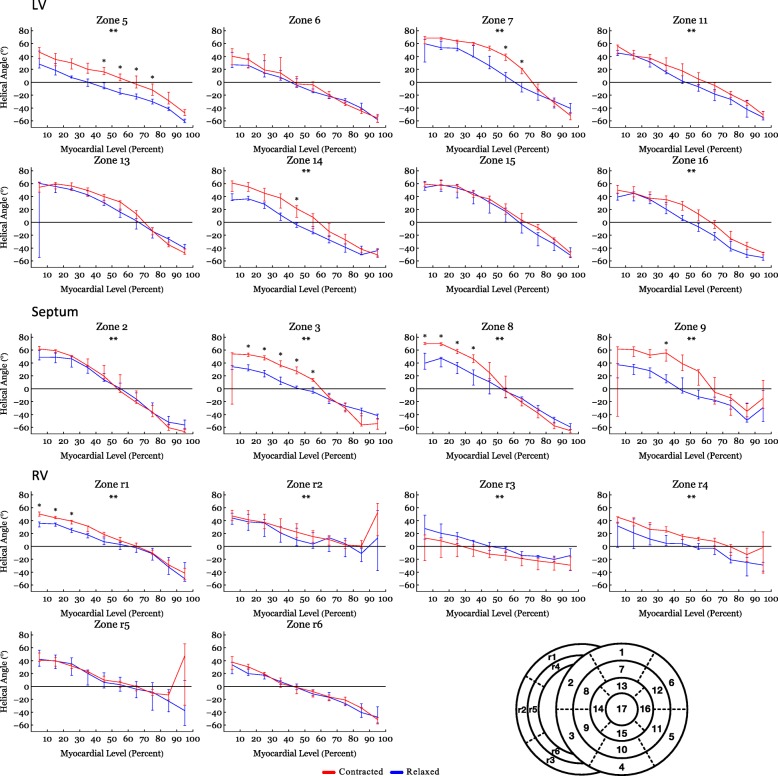
Angles versus myocardial depth – Helical angle sub-analysis. Helical angles as assessed in the individual subzones as a function of myocardial depth in percent. 0% is the sub-endocardium, 100% is the sub-epicardium. In the interventricular septum 0% is the left ventricular sub-endocardium and 100% is the right ventricular sub-endocardium. Data are shown as medians (Interquartile Range). **: Statistically significant overall two-way ANOVA. *: Statistical significance in the individual area after Bonferroni correction

**Fig. 8:**
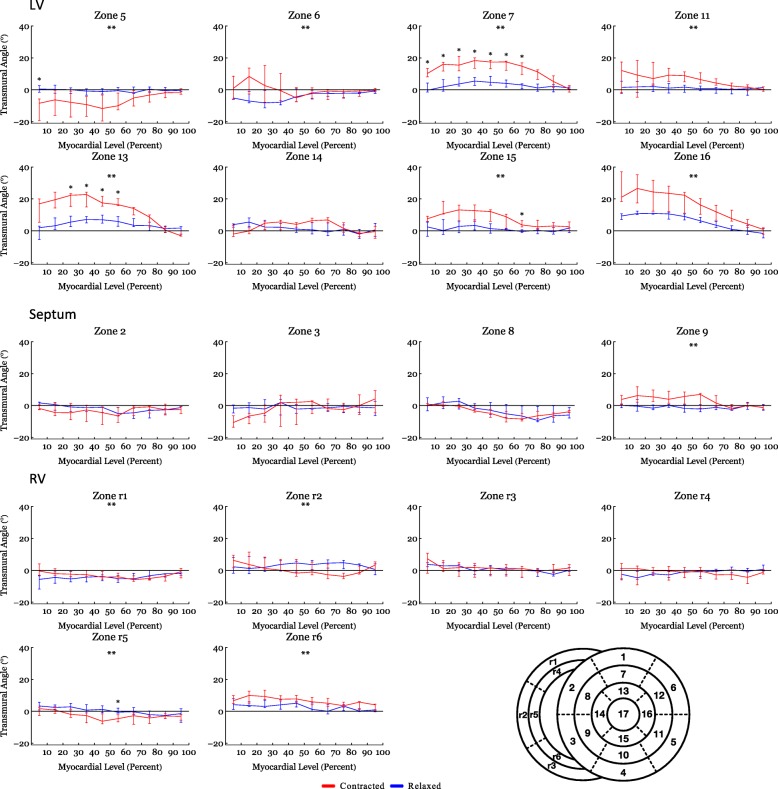
Angles versus myocardial depth – Transmural angle sub-analysis. Transmural angles as assessed in the individual subzones as a function of myocardial depth in percent. 0% is the sub-endocardium, 100% is the sub-epicardium. In the interventricular septum 0% is the left ventricular sub-endocardium and 100% is the right ventricular sub-endocardium. Data are shown as medians (Interquartile Range). **: Statistically significant overall two-way ANOVA. *: Statistical significance in the individual area after Bonferroni correction

**Fig. 9:**
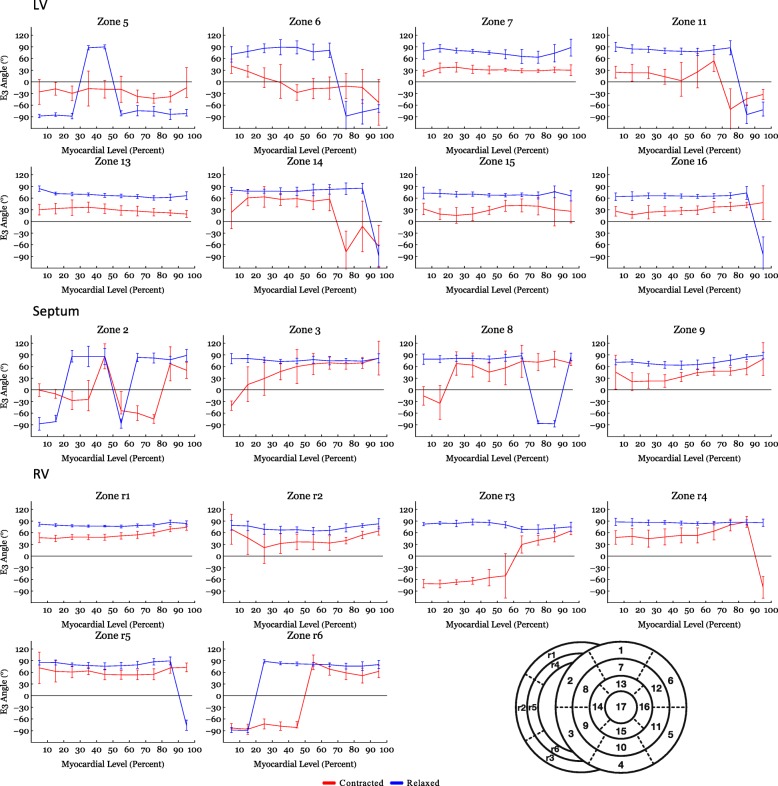
Angles versus myocardial depth – E3 sub-analysis. E3-angles in the individual subzones as a function of myocardial depth in percent. 0% is the sub-endocardium, 100% is the sub-epicardium. In the interventricular septum 0% is the left ventricular sub-endocardium and 100% is the right ventricular sub-endocardium. Data are shown as circular means (95% Confidence Intervals). Asterisks indicate statistical significance in the individual area after Bonferroni Correction

**Fig. 10:**
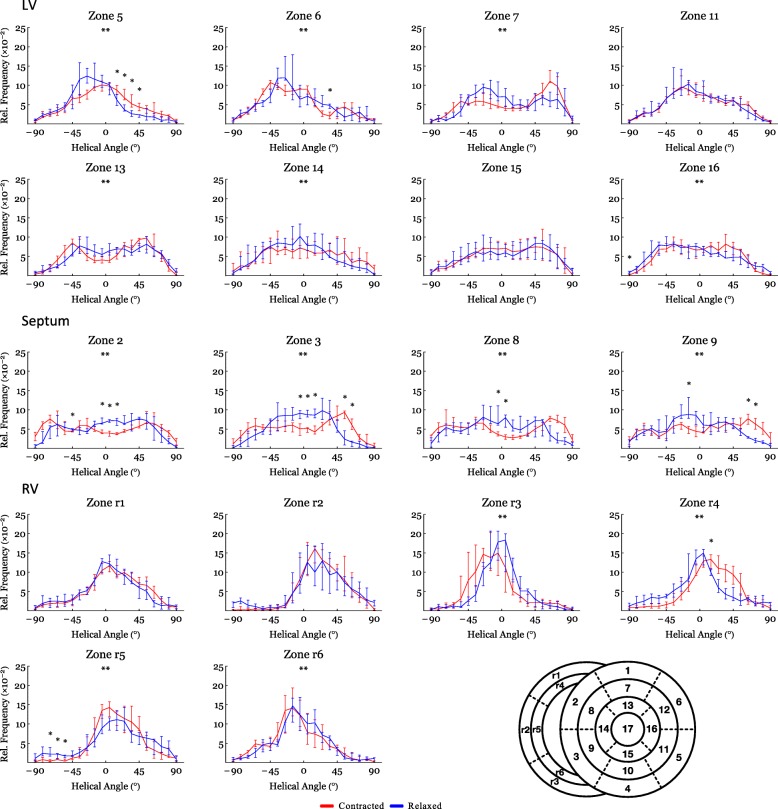
Angle distributions – Helical sub-analysis. Histograms of the helical angle distribution from relaxation to contraction in the individual sub-zones. Data are shown as medians (Interquartile Range). **: Statistically significant overall two-way ANOVA. *: Statistical significance in the individual area derived from post-hoc testing with Mann-Whitney U-Test with Bonferroni correction

**Fig. 11:**
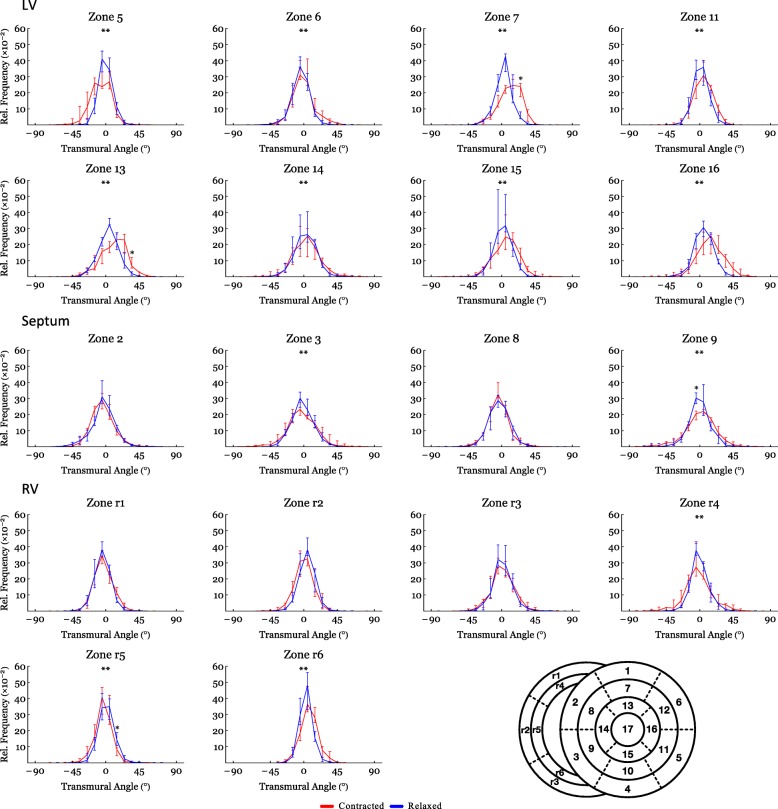
Angle distributions – Transmural sub-analysis. Histograms of the transmural angle distribution from relaxation to contraction in the individual sub-zones. Data are shown as medians (Interquartile Range). **: Statistically significant overall two-way ANOVA. *: Statistical significance in the individual area derived from post-hoc testing with Mann-Whitney U-Test with Bonferroni correction

**Fig. 12:**
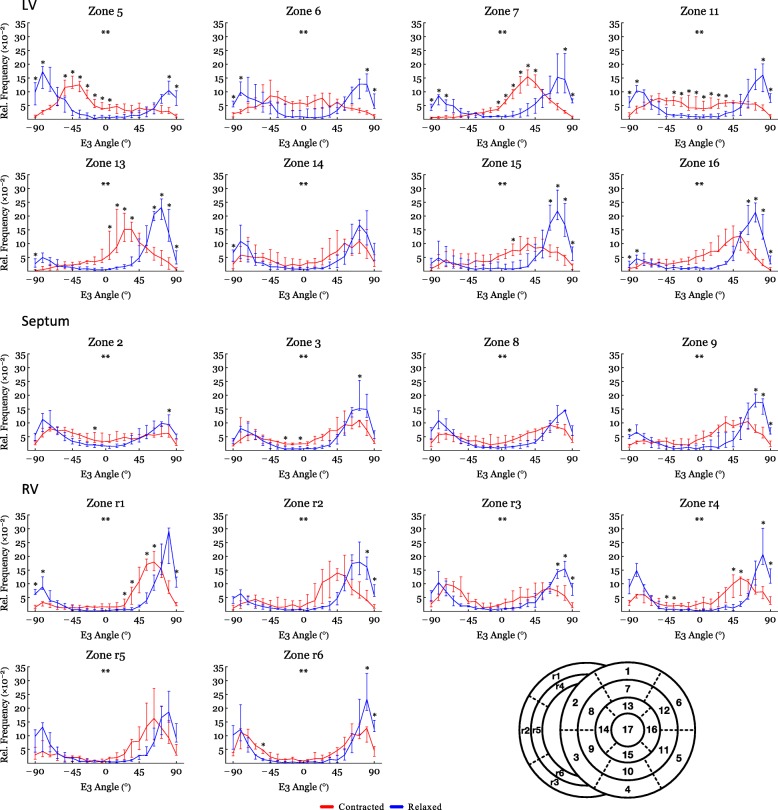
Angle distributions – E3 sub-analysis. Histograms of the E3-angle distribution from relaxation to contraction in the individual sub-zones. Data are shown as medians (Interquartile Range). **: Statistically significant overall two-way ANOVA. *: Statistical significance in the individual area derived from post-hoc testing with Mann-Whitney U-Test with Bonferroni correction

Figure 4 shows histograms of the helical, transmural and E3-angle distribution in the relaxed state versus the contracted state for the entire LV, RV and septum. Figure 5 shows plots of helical, transmural and E3-angles as a function of mural thickness, setting the endocardium to 0% and epicardium to 100%, as originally proposed by Streeter and colleagues [23]. Angle colour maps from the equatorial level are provided in Fig. 6. Table 2 shows the post-hoc analysis of the helical, transmural and E3-angles based on myocardial depth. Plots of the helical, transmural and E3-angles in each individual zone both shown as a function of mural thickness and as histograms are provided in Figs. 7, 8, 9, 10, 11 and 12.

**Table 2:** Post-hoc analysis of angle data based on myocardial depth

	Endocardium (0–33%)			Midwall (33–66%)			Epicardium (66–100%)		
	Relaxed state	Contracted state	*P*-value	Relaxed state	Contracted state	*P*-value	Relaxed state	Contracted state	*P*-value
The Helical Angle									
Left Ventricle	35.4 (23.4;53.2)	47.8 (32.4;53.8)	0.051	−2.4 (−10.7;16.8)	16.0 (−0.9;27.1)	< 0.001	−35.3 (−40.8;-27.7)	−32.4 (− 37.4;-26.3)	NS
Septum	34.0 (27.4;44.0)	56.5 (51.0;61.4)	< 0.001	2.0 (−7.2;9.1)	19.1 (10.5;34.)	< 0.001	−36.3 (−44.8;-29.5)	−40.0 (−51.8;-30.4)	NS
Right Ventricle	25.9 (16.5;37.3)	32.0 (24.8;41.8)	NS	0.1 (−4.7;9.0)	8.9 (−2.2;14.1)	NS	−18.1 (− 28.8;-6.9)	−14.0 (− 25.6;-1.3)	NS
Entire Heart	30.8 (21.8;43.9)	45.5 (30.6;54.2)	NS	−0.2 (−7.9;11.9)	13.7 (−0.5;25.3)	NS	−30.6 (− 39.5;-19.8)	−29.7 (−37.5;-17.8)	NS
The Transmural Angle									
Left Ventricle	3.1 (−1.7;7.)	10.4 (−1.1;18.3)	0.007	1.2 (−0.9;6.6)	5.6 (−0.7;13.7)	0.013	−0.5 (−2.2;3.0)	1.0 (−2.0;4.8)	NS
Septum	−0.2 (−2.8;2.6)	0.2 (−5.1;2.5)	NS	−2.2 (−4.4;-0.1)	− 2.1 (−7.6;3.8)	NS	−2.2 (− 5.6;-0.7)	− 1.9 (− 5.1;-0.1)	NS
Right Ventricle	0.7 (− 2.4;4.1)	1.8 (− 2.5;5.4)	NS	− 0.4 (− 2.1;3.7)	−1.6 (− 5.0;3.7)	NS	− 0.6 (− 2.0;1.8)	−1.5 (− 4.2;1.6)	NS
Entire Heart	1.0 (−2.4;5.8)	2.3 (− 2.8;10.6)	NS	− 0.2 (− 2.5;4.0)	0.9 (−5.0;7.7)	NS	−0.8 (− 2.4;1.6)	−0.4 (−3.6;2.9)	NS
The E3-Angle									
Left Ventricle	78.6 (75.3;81.8)	24.8 (17.1;32.4)	< 0.001	76.2 (72.2;80.2)	25.3 (15.0;35.7)	< 0.001	80.8 (74.5;87.2)	9.6 (−12.4;31.6)	< 0.001
Septum	79.7 (74.4;85.1)	6.3 (−13.7;26.4)	< 0.001	76.8 (71.2;82.4)	54.2 (36.0;72.3)	< 0.001	82.0 (77.6;86.5)	66.0 (56.9;75.1)	< 0.001
Right Ventricle	82.6 (79.6;85.7)	68.6 (51.9;85.3)	< 0.001	78.0 (75.0;81.0)	58.4 (47.0;69.8)	< 0.001	80.0 (76.6;83.5)	60.4 (54.3;66.4)	< 0.001
Entire Heart	80.2 (78.1;82.3)	31.9 (23.0;40.9)	< 0.001	77.0 (74.6;79.3)	42.4 (34.5;50.4)	< 0.001	80.8 (77.9;83.8)	53.6 (45.4;61.8)	< 0.001

Helical and transmural angles are shown as medians (Interquartile Range) while E3-angles are shown as circular means (CI). NS, statistically non-significant. Groups are compared using Mann-Whitney U-test

**Fig. 13:**
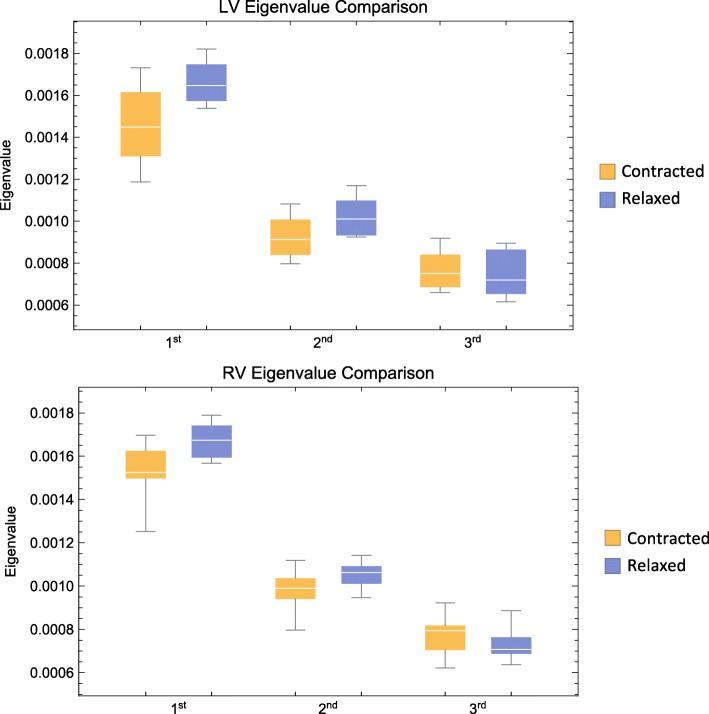
Eigenvalue comparison. Comparison of the three eigenvalues in the left ventricle. Box plot of eigenvalues of the first, secondary and tertiary eigenvectors. Means are calculated from all voxels in each individual heart. The plots are subsequently generated from summarizing the individual means within group. Boxes span the distance between the 0.25 and 0.75 quantiles surrounding the median with whiskers that span the full dataset

Furthermore, eigenvalue comparison of the LV and the RV can be appreciated in Fig. 13, showing significant difference between all eigenvectors in both ventricles both in the contracted state and in the relaxed state.

#### Helical angle

In Fig. 4 it is shown that from relaxation to contraction the helical angle distribution differed significantly, in the LV wall (*p* = 0.02) and the RV wall (*p* = 0.004). In the septum, however, an even more obvious change in the distribution of helical angles was seen from relaxation to contraction (*p* < 0.001). Relative to myocardial wall depth helical angles changed significantly from relaxation to contraction in the LV and in the septum, but not in the RV (Fig. 5). In the LV, the median helical angle changed from 35.4° in the relaxed state to 47.8° in the contracted state (*p* = 0.051) in the endocardium (Table 2). In the midwall the angle increased from − 2.4° in the relaxed state to 16.0° in the contracted state (*p* < 0.001). In the epicardium the mean helical angle did not change significantly from relaxation to contraction. No significant changes of the helical angles were found from relaxation to contraction in the RV myocardium as a whole. If, however, each zone of the RV myocardium was considered individually, significant changes in helical angles were found in several zones of the RV (Fig. 7). In the septal endocardium (of the LV) the median helical angle increased from 34.0° in the relaxed state to 56.5° in the contracted state (*p* < 0.001) and in the midwall an increase was found from 2.0° in the relaxed state to 19.1° in in the contracted state (*p* < 0.001), see Table 2.

#### Transmural angle

In both ventricles and in the septum, the proportion of transmural angles with a value close to zero decreased from relaxation to contraction. From Fig. 4 it is, furthermore, noteworthy that individual transmural angles approach, and in the septum even supersede, 45°. When assessing the change in transmural angle from relaxation to contraction across the wall significant changes were found only in the LV (Fig. 5). Here the median transmural angle increased from 3.1° in the relaxed state to 10.4° in in the contracted state in the endocardium (*p* = 0.007) and in the midwall this angle increased from 1.2° in the relaxed state to 5.6° in the contracted state (*p* = 0.013). No significant change was found in the epicardium of the LV (Table 2).

An assessment of the transmural angle change in the individual subzones can be appreciated as a function of myocardial depth in Fig. 8 and as histograms in Fig. 11. As stated above no significant transmural angle change was found in either the septum or the RV when assessed as a function of myocardial depth. Significant changes, however, were found within more of the subzones in the LV (Fig. 8).

#### E3-angle

Significant differences in the distribution of E3-angles from relaxation to contraction were found in all parts of the heart (Fig. 4). Figure 5 shows that all parts of the heart exhibited significant decreases of the E3-angle, when plotted against of myocardial level.

In Table 2 we show that in the LV, the mean E3-angle decreased from 78.6° in the relaxed state to 24.8° in the contracted state in the endocardium (*p* < 0.001). In the midwall the E3-angle decreased from 76.2° in the relaxed state to 25.3° in the contracted state (*p* < 0.001) and in the epicardium a decrease was found from 80.8° in the relaxed state to 9.6° in the contracted state (*p* < 0.001). In the RV, the E3-angle decreased from 82.6° in the relaxed state to 68.6° in the contracted state in the endocardium (*p* < 0.001). In the midwall a decrease from 78.0° in the relaxed state to 58.4° in the contracted state was seen (*p* < 0.001) and in the epicardium a decrease was found from 80.0° in the relaxed state to 60.4° in the contracted state (*p* < 0.001). In the septum the E3-angle decreased from 79.7° in the relaxed state to 6.3° in the contracted state in the endocardium and in the midwall a decrease from 76.8° in the relaxed state to 54.2° in the contracted state was found (*p* < 0.001). In the epicardium a decrease was found from 82.0° in the relaxed state to 66.0° in the contracted state (*p* < 0.001).

## Discussion

This is the first study describing the dynamic changes in the orientation of the cardiomyocytes within the RV myocardium between two contractional states. We, moreover, confirm existing knowledge on the dynamic changes of the myocardial architecture, that accompany LV deformation [2, 18, 20]. Previous in-vivo studies have shown that myocardial deformation of the LV is mainly driven by rearrangement of the aggregations of cardiomyocytes [20].

In the RV, the proportion of E3-angles approaching 0° increased from relaxation to contraction as the aggregates assume a more radial or horizontal orientation in the contracted state. Similarly, in the LV, a shift towards smaller E3-angles was observed in the contracted state, which is in line with previous in-vivo studies [2, 18, 20]. No significant change in neither the helical nor the transmural angulation was found in the RV myocardium as a whole. However, when assessing each RV myocardial zone, individually significant changes in the helical angle were found locally (Fig. 7). The transmural angulation, however, remained unchanged in the RV also when assessed in individual zones (Fig. 8). These findings are very interesting in light of the newly formed hypotheses of intrinsic myocardial antagonism [28–30]. As no change in transmural angulation is found in the RV, this could imply that the need for a very refined contraction, mediated by antagonistic forces, is not as prominent in the RV as in the LV.

Few have investigated RV micro-architecture using diffusion tensor imaging; this may be due to the fact that the importance of the RV has been underestimated for many years [9], but it may also be because diffusion tensor magnetic resonance imaging previously offered insufficient spatial resolution to permit valid investigation of the thin-walled RV.

The present study shows no change in transmural angulation and only discrete changes in helical angle in the RV. But what mechanisms are then facilitating wall deformation from diastole to systole in the RV? Ejection of blood from the ventricles is provided by a combination of circumferential constriction and longitudinal shortening of the ventricle resulting in myocardial wall thickening [31]. To achieve this the constant myocardial mass must be repacked [32]. Previous studies on the LV indicate that the aggregations of cardiomyocytes are the predominant mediator of myocardial wall thickening [20]. When considering Fig. 4 the rearrangement of the E3-angle shows a similar pattern in both ventricles of the heart and in the interventricular septum. The current study, therefore, suggests that changes in the orientation of the aggregates is the predominant mediator of myocardial wall thickening in the entire heart and not just in the LV.

Our observations provide new insights into the dynamic differences between the various parts of the heart. As mentioned in the introduction, the two ventricles differ in many aspects although serving the same overall purpose. Most notably, the gross anatomy differs greatly. The RV has a very complex shape often referred to as a flattened tube wrapped around the LV [33], which leads to the implication that the septum is a LV structure. This notion has been presented in literature for centuries [34, 35]. Recently, this concept of septal ownership has been questioned based on investigations using diffusion tensor imaging [22]. The present study adds further to this discussion because it appears the dynamic myocardial remodelling of the septum differs from that of the remainder of the myocardium suggesting that myocardial deformation is very heterogeneous across the heart. The functional significance of these findings remains speculative, but from an anatomical point of view our data confirm the notion, that the septum cannot solely be attributed a LV structure, as has often been the case in literature.

In addition to the anatomical differences described above, RV physiology differs markedly from that of the LV [4, 33]. In the foetal circulation the RV and the LV are of equal hemodynamic importance. In this setting the ventricular wall-thicknesses are equal as both ventricles work against approximately similar afterloads. After birth, dramatic physiological remodelling occurs in the circulation of the infant, and the LV must generate a higher pressure in order to serve the need from the systemic circulation. The RV on the other hand pumps against little resistance, and the physiological hypertrophy of the RV myocardium regresses leaving the wall 3–4 times thinner than the LV [9, 33]. It could be argued that the difference in myocardial wall-thickness explains the functional difference between the two ventricles. If, however, a child is born with a functionally univentricular heart, the morphological origin of the systemic ventricle is of paramount importance. The survival rate is significantly worse if born with a dominant RV compared with being born with a dominant LV in spite of the ventricular transmural thickness being equal in both cases [36]. It seems, therefore, that the different performance of the ventricles in this setting cannot be contributed to differences in mural thickness, but to different intrinsic properties within the LV and the RV myocardium.

### Study limitations

The most relevant limitation to this present study is the lack of dynamic in-vivo diffusion tensor imaging. This has previously been performed on the human LV [19, 20]. In the present study, however, spatial resolution was of paramount importance in order to obtain data of sufficient quality from the thin myocardium of the RV. As it is not possible to achieve this in-vivo at present, ex-vivo diffusion tensor imaging was the method of choice.

According to our wall thickness comparison (Fig. 3), some parts of the heart appear to be in a hypercontracted state. This is most pronounced in the LV and could potentially cause exaggeration of the angle differences within these regions. As stated above our LV findings are, however, comparable to previous studies on the dynamic changes of the myocardial architecture, that accompany LV deformation [2, 18, 20]. The novelty of this study lies within the RV results. In this setting it is noteworthy that the RV and the septal contraction appear close to and within the physiological range.

The remodelling of the RV myocardium is a novel field of research, and it can be argued that diffusion tensor imaging is only histopathologically validated in the LV. The technique has, however, been validated several times over the years using both two-dimensional and three-dimensional techniques, thus we consider the accumulated evidence of the validity of the technique very convincing and see no reason why it should not likewise apply to the RV [37–39].

In addition, it can be argued that ventricular volumes should be used when comparing the contractional states. We were, however, facing the challenge of comparing low-resolution in-vivo data with high-resolution ex-vivo data. This introduced a considerable inaccuracy in the comparison, which increases for each dimension assessed. We are, therefore, reducing the risk of a type two error by using ventricular wall thickness as a measure of state of contraction.

Further, we used the E3-angle as a pseudo-marker of the orientation of the aggregates of cardiomyocytes. This angle is assessed using the tertiary eigenvector, which is a mathematical derivative of the primary and secondary eigenvectors and is hence not a direct depiction of the actual structure. From Fig. 13 we see a significant difference between the first, secondary and tertiary eigenvector meaning that distinction between the secondary eigenvector and the tertiary eigenvector is valid. The widespread use of different names for the aggregations is an expression of poor understanding of the actual three-dimensional anatomical extent of these aggregates. There is, however, agreement in literature that the aggregates are of a planar nature. The mathematically correct way of assessing the orientation of a plane in space is by the use of its normal vector. We thus consider the use of the E3-angle not only justified, but also more accurate than the use of any other previously suggested alternative.

## Conclusion

The architecture of the entire myocardium changes through the cardiac cycle. This rearrangement is provided by continuous changes in the orientation of cardiomyocytes. The movement of cardiomyocytes is, however, different in the RV and LV and in the septum, potentially explaining the different physiological capabilities of the various parts of the heart. The current study provides new insight into the micro-structural mechanisms underlying RV contraction, suggesting that the change in the orientation of the aggregates of cardiomyocytes, quantified by the E3-angle, is the predominant mediator of myocardial wall thickening not only in the LV, but in the entire myocardium. This study also provides high-resolution validation of recent LV in-vivo measurements, paving the way for potential diagnosis of heart disease based on the function of ventricular micro-structure.

## Additional file

Additional file 1: Rotatable 3D PDF showing the angle definitions. The local set of orthogonal planes for the assessment of cardiomyocyte orientation. The plane A is the local epicardial tangential plane and plane C is defined as the plane spanned by normal of plane A and the local epicardial horizontal (short axis) tangent. Plane B is orthogonal to planes A and C. The helical angle is defined as the angle between the cardiomyocytes (yellow rod) and plane C. The transmural angle is defined as the angle between the cardiomyocytes and the epicardial tangential plane A. The E3-angle is defined as the angulation between the aggregate plane (red box) and the epicardial tangential plane A. (PDF 100 kb)

## Data Availability

The datasets used and/or analysed during the current study are available from the corresponding author on reasonable request.

## Abbreviations

CMR: Cardiovascular magnetic resonance
LV: Left ventricle/left ventricular
RV: Right ventricle/right ventricular
