# Supplementary figures and images for: Resolving the natural myocardial remodelling brought upon by cardiac contraction; a porcine ex-vivo cardiovascular magnetic resonance study of the left and right ventricle
Source: J Cardiovasc Magn Reson. 2019 Jul 1;21:35. doi: 10.1186/s12968-019-0547-2 (PMC6600899; doi:10.1186/s12968-019-0547-2)

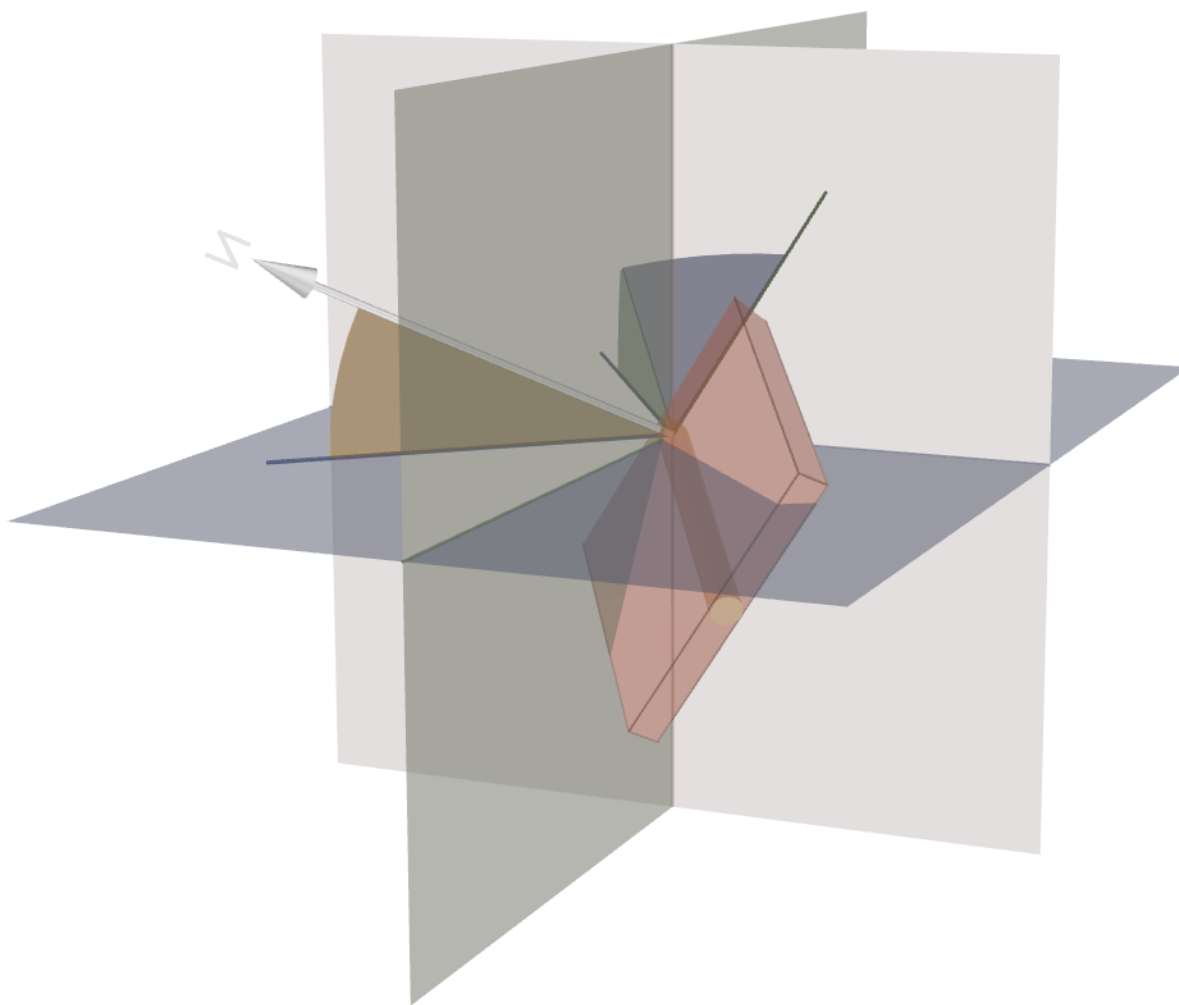

Supplement: Supplementary file 1 — Rotatable 3D PDF showing the angle definitions. The local set of orthogonal planes for the assessment of cardiomyocyte orientation. The plane A is the local epicardial tangential plane and plane C is defined as the plane spanned by normal of plane A and the local epicardial horizontal (short axis) tangent. Plane B is orthogonal to planes A and C. The helical angle is defined as the angle between the cardiomyocytes (yellow rod) and plane C. The transmural angle is defined as the angle between the cardiomyocytes and the epicardial tangential plane A. The E3-angle is defined as the angulation between the aggregate plane (red box) and the epicardial tangential plane A. (PDF 100 kb) [file 12968_2019_547_MOESM1_ESM.pdf]
